# Sensor Fusion of Monocular Cameras and Laser Rangefinders for Line-Based Simultaneous Localization and Mapping (SLAM) Tasks in Autonomous Mobile Robots

**DOI:** 10.3390/s120100429

**Published:** 2012-01-04

**Authors:** Xinzheng Zhang, Ahmad B. Rad, Yiu-Kwong Wong

**Affiliations:** 1 School of Electrical and Information Engineering, Jinan University, Zhuhai 519070, Guangdong, China; E-Mail: ee.xz.zhang@gmail.com; 2 School of Engineering Science, Mechatronic System Engineering, Simon Fraser University, 250-13450, 102 Avenue, Surrey, BC, V3T 0A3, Canada; 3 Department of Electrical Engineering, The Hong Kong Polytechnic University, Hung Hom, Kowloon, Hong Kong; E-Mail: eeykwong@polyu.edu.hk

**Keywords:** feature fusion, multi-sensor point estimation fusion (MPEF), homography transform matrix, SLAM

## Abstract

This paper presents a sensor fusion strategy applied for Simultaneous Localization and Mapping (SLAM) in dynamic environments. The designed approach consists of two features: (i) the first one is a fusion module which synthesizes line segments obtained from laser rangefinder and line features extracted from monocular camera. This policy eliminates any pseudo segments that appear from any momentary pause of dynamic objects in laser data. (ii) The second characteristic is a modified multi-sensor point estimation fusion SLAM (MPEF-SLAM) that incorporates two individual Extended Kalman Filter (EKF) based SLAM algorithms: monocular and laser SLAM. The error of the localization in fused SLAM is reduced compared with those of individual SLAM. Additionally, a new data association technique based on the homography transformation matrix is developed for monocular SLAM. This data association method relaxes the pleonastic computation. The experimental results validate the performance of the proposed sensor fusion and data association method.

## 1. Introduction

A crucial characteristic of an autonomous mobile robot is its ability to determine its whereabouts and make sense of its static and dynamic environments. The central question of perception of its position in known and unknown world has received great attention in robotics research community. Mapping, localization, and particularly their integration in the form of Simultaneous Localization and Mapping (SLAM) is the basic ability with which other advanced tasks such as exploration and autonomous navigation can be successfully implemented. Therefore, SLAM has been vigorously pursued in the mobile robot research field.

Monocular cameras have been widely used as low cost sensors in numerous robotics applications in recent years. They provide the autonomous mobile robot with abundant information that facilitates intuitive interpretation and comprehension of the environment better than other scanning sensors. The algorithms based on monocular cameras perform reasonable visual SLAM procedures. Points/landmarks extracted from images are common map elements and typically present in structured indoor scenes [1–5]. However, they are easily occluded in dynamic environments if sufficient precautions are not devised. Features of the segment-based map consist of lines and edges which are stable compared with point features and consequently robust enough to improve the performance of the monocular SLAM [6–9]. Most of relevant research above, however, implemented SLAM in static spaces or environments with few moving objects. Dynamic objects induce spurious features and make it difficult to obtain the correct estimates of the robot pose and feature positions. Additionally, erroneously extracted map features corresponding to dynamic objects may lead to inappropriate robot actions that ultimately result in failure to complete the expected tasks.

In this study, we present a sensor fusion strategy for line-based SLAM applied in dynamic environments. This approach fuses the sensor information obtained from a monocular camera and laser rangefinder and includes two modules. One is a feature fusion that integrates the lines extracted respectively from a monocular camera and a laser to remove the erroneous features corresponding to dynamic objects. The other is referred to as a modified multi-sensor point estimation fusion SLAM (MPEF-SLAM) which incorporates two separate EKF SLAM frameworks: monocular and laser SLAM. This modified MPEF-SLAM fuses the state variable and its covariance estimated from individual SLAM procedure and propagates fused values backward to each SLAM process to reduce the error of robot pose and line feature positions. Another advantage of the modified MPEF-SLAM is that its implementation is on the basis of two parallel running SLAM processes, which can avoid unexpected events. For example, when one SLAM procedure does not work due to the sensor failure, the other one can be still running normally. This manifests the idea of redundancy in comparison with the single SLAM framework. Additionally, for monocular SLAM process we suggest a new data association (DA) algorithm. It employs the homography transformation matrix [10] estimated by the matched points determined through Scale Invariant Feature Transform (SIFT) descriptors [11] in two images. The sensor fusion strategy is examined and tested in practical experiments. The results demonstrate that the proposed approach can reliably filter out dynamic aspects and yields accurate models of the environment, as well enhance the localization precision.

The remainder of this paper is organized as follows: after reviewing the related work in Section 2, we elucidate the framework of monocular SLAM and the data association method in Section 3. Section 4 describes the sensor fusion strategy including the feature fusion and modified MPEF-SLAM modules. We validate our proposed methods through the experiments in Section 5. Section 6 gives our conclusions and suggestions for future work.

## 2. Related Work

Most indoor environments can simply and expediently be represented by line segments. In our previous work [12] and references therein, the line features have been successfully applied to various SLAM algorithms by range scanner sensors. In recent decades, advances in computer vision have provided robotics researchers with efficient and powerful techniques that can be employed in a variety of autonomous tasks. Following Davison and his group’s pioneering work on monocular SLAM [1–5], other researchers studied line-based algorithms. Eade and Drummond [6] proposed an edge-let landmark to depict the line features. This work, which is the extension of the so-called scalable monocular SLAM [4], avoids regions of conflict and deals with multiple matches through robust estimation. Gee and Mayol-Cuevas [7] used fast conic extraction to obtain the 2D edges and then estimated the 3D segments with the Unscented Kalman filter (UKF). Smith *et al*. [8] applied FAST corners to quickly verify that there was an edge between two corners by bisecting checks. Besides, other researchers conducted similar studies on line based SLAM with a single camera. Lemaire and Lacroix [9] as well as Sola *et al*. [13] introduced the Plücker coordinates for 3D line description and considered constraints associated with Plücker representation during the updating stage of Kalman filter. Folkesson *et al*. [14] suggested a M-space feature representation similar to SP-model. This feature model is a general and systematic technique that makes it possible to change sensors and features without any variation to SLAM implementation. In addition, lines and points can be merged to enhance the performance of visual SLAM and improve the precision of the localization and mapping [15,16]. The vertical and the floor lines can also be combined to represent the environment in a more complete fashion via a unified EKF framework by integrating two different measurement models [17].

To the best of our knowledge, almost all mentioned methods above focus on the visual SLAM in static space or the environments with few dynamic objects. In this study, we re-visit the SLAM problem in a dynamic environment from a sensor fusion viewpoint. This approach incorporates the sensor information of a monocular camera and laser rangefinder to remove the feature outliers related to dynamic objects and enhances the accuracy of the localization.

Computer vision technology makes visual SLAM feasible, and related data association methodology is also an interesting area which has attracted much research attention. In addition to conventional data association algorithms such as Nearest Neighbor [7,8], JCBB [18], *etc*., several data association methods for visual SLAM has been developed, including Normalized Cross-Correlation (NCC) [19,20], incremental expectation maximization algorithm [21], incremental hierarchical data association based on image similarity [22], homography tracking [23] and those based on the SIFT descriptor. The invariant property of the SIFT descriptor is an important factor for the SIFT based data association method. For example, in [24,25] landmarks are identified by SIFT and represented by keypoint descriptors. These landmarks subsequently are treated as the ideal candidates for robust data association. Gil *et al*. [26,27] managed the data association with the SIFT features from the pattern classification viewpoint, and the Mahalanobis distance was established by the average SIFT descriptors and a high dimensional covariance matrix. Similarly with pattern recognition technology, object-based SLAM [28] combined the advantages of multi-scale Harris corner detector and the SIFT descriptor for natural object recognition, which provides a correct data association. Also, to enhance the robustness of SIFT descriptor a multi-resolution descriptor was proposed to address the problem that the performance gains diminish when uncertainty about camera position increases [29].

These data association methods using the SIFT technique improve the robustness compared with the NCC and image patches based approaches. With this advantage, we also developed a data association method that does not apply the SIFT descriptor as the map features but rather uses the descriptor to estimate the homography transformation matrix of any two images, and then employs the estimated homography transformation matrix to implement data association.

## 3. Line Based EKF Monocular SLAM

In this section the outline of the line-based monocular SLAM framework is discussed. We briefly present the camera/robot motion and line measurement model. After that the homography transformation matrix based data association method for monocular SLAM is introduced.

### 3.1. Camera/Robot Motion Model and Line Measurement Model

**Figure 1.**
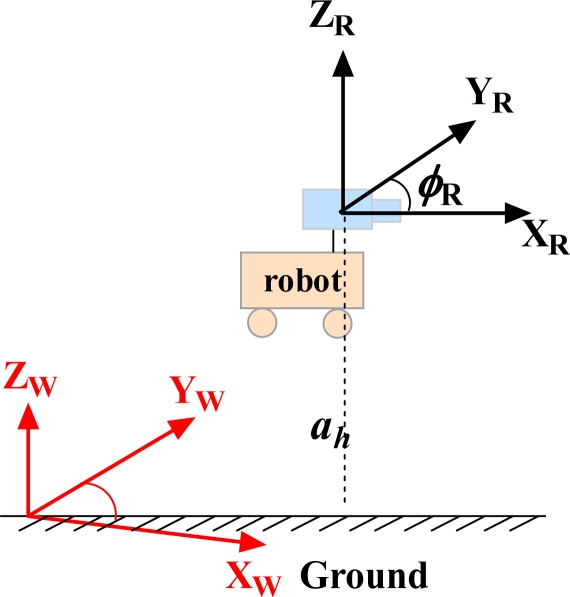
The world and robot/camera coordinate reference. Red and subscript W: world reference; black and subscript R: camera/robot reference. *a_h_* is the height from the ground plane.

The camera is fixed on the robot platform which moves in a 2D plane, and its translational and rotational velocity are identical with the mobile robot. For convenience, as is shown in Figure 1 we assume the origins of the robot and the camera reference frames are located at the same point.

The simplified camera motion model is:

(1) $$x_{v(k)}=\left(\begin{array}{l} x_{R(k)}\\ y_{R(k)}\\ \phi_{R(k)} \end{array}\right)=f_{v}\left(x_{v(k-1)},u_{k}\right)=\left(\begin{array}{l} x_{R(k-1)}+v_{k}\;\text{cos}\;\phi_{k}\Delta t\\ y_{R(k-1)}+v_{k}\;\text{sin}\;\phi_{k}\Delta t\\ \phi_{R(k-1)}+\omega_{k}\Delta t \end{array}\right)$$

where **x**_*v*(*k*)_ is the robot pose at time *k* including the position (*x_R_*_(_*_k_*_)_, *y_R_*_(_*_k_*_)_)*^T^* and head orientation *ϕ_R_*_(_*_k_*_)_; **u***_k_* is the control variable at time *k* including the translation velocity *v_k_* and rotational velocity *ω_k_*; Δ*t* is the sampling time.

**Figure 2.**
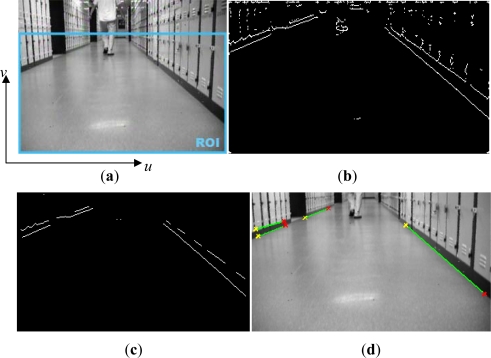
(**a**) The region of interest (ROI); (**b**) Detected horizontal edges in ROI without morphological operation, and some edges related to dynamic objects are not removed; (**c**) Detected horizontal edges in ROI after *shrink* and *clean* morphological operation with *thresholding* technique; (**d**) Selected line features in ROI after *thicken* operation.

Line extraction is actually an edge detection operation in the image processing terminology. Most of the edge features represented in the related work are extracted by using a first-order edge detection operator: the canny operator [6,7]. In this current study, we employed another first-order edge detector: the Sobel operator combined with a thresholding technique for edge extraction in a specified region of interest (ROI) displayed in Figure 2(a).

The range of ROI encapsulates the ground plane since most static edges are present on the floor. In this ROI we just consider the horizontal static edges, and do not focus on tracking the dynamic targets. After horizontal edge detection processing, it is clear that several edges corresponding to the dynamic objects (*i.e.*, the person here) cannot be eliminated from the selected region, which is illustrated in Figure 2(b). To reduce the effect of these potential outliers, the *shrink* and *clean* morphological operations firstly are carried out on all edges. With these operations, the shorter and thinner edges, which usually relate to the parts of dynamic objects, are removed; and secondly for the rest of edges, if the length of an edge is less than the length threshold (in pixels) it is also rejected from the edges set. This operation makes sure to further exclude spurious edges not removed in *shrink* and *clean* process (cf. Figure 2(c)). Finally the *thicken* operation is implemented to recover the interested edges and highlight them (cf. Figure 2(d)), which will prepare for edge parameter extraction in the next step.

We developed two sets of parameters for edge representation: one was used in the measurement model; the other was for data association and sensor fusion. In this subsection, we mainly discuss the parameters for the measurement model. For a line reflected in the vision system, the minimal representation uses four parameters (e.g., Denavit-Hartenberg line coordinates) in 3D Euclidean space but it may be ineffective in some robotic research topics. There are several non-minimal representations for the 3D line, such as endpoints of the line [8], center and unit direction vector of the line [6], two endpoints plus unit direction vectors [7], and so on. In this study, we also describe the lines by the line endpoints non-minimal representation because the advantages are this representation is homogenous and suitable for the projection through a pinhole camera.

Similar to [7,8], with the location of line endpoints we borrowed and extended the idea of their work and presented the line measurement model as:

(2) $${\mathrm{pt}}_{\mathrm{mk}}=d_{k}^{m}R_{M\;k}{\hat{e}}_{k}^{m}+x_{\mathrm{vk}}$$

(3) $$\left(\begin{array}{l} h_{k}\\ \varphi_{k} \end{array}\right)=f\left({\mathrm{pt}}_{0k},{\mathrm{pt}}_{1k}\right)$$

(4) $$z_{k}={\left(\begin{array}{ccc} p_{\mathrm{ek}} & \varpi_{k} & p_{\mathrm{ipk}} \end{array}\right)}^{T}=\Pi \left({\mathrm{pt}}_{0k},{\mathrm{pt}}_{1k},h_{k},\varphi_{k},P_{\mathrm{ck}}\right)+n_{k}$$

where at step *k*, in Equation (2)**pt***_mk_* is the 3D location of the *m*th endpoint in the world coordinate system, $d_{k}^{m}$ is the depth of the *m*th endpoint from the camera center, **R**_**M***k*_ is the rotation matrix associated with quaternion, and ${\hat{e}}_{k}^{m}$ is the unit vector direction of the *m*th endpoint from the camera center of projection; in Equation (3)*h_k_* is the length of the normal and *ϕ_k_* is the angle of inclination of the normal from the camera/robot framework which will be used for feature fusion, *f* is the function in matrix form for calculating *h_k_* and *ϕ_k_*; Equation (4) computes the parameters of the 2D line in the image plane. These parameters consist of the measurement variables, including the orientation *ϖ_k_* of the 2D line, the locations of the line’s endpoints *p_ek_* (*i.e.*, pixel coordinates), and coordinates of the projection which is the intersection point *p_ipk_* between a 3D line and the normal of the line (projected intersection point for short in the following sections). Π is the standard pin-hole projection function for a calibrated camera, **P***_ck_* is the mean camera projection matrix estimated in the *k*th step, and ***n****_k_* is the measurement noise from the image. To make a simple presentation, we define the pixel coordinates of the line endpoints *p_ek_* and projected intersection point *p_ipk_* in a unified form *i.e.*, *p* = (*x_u_*,*y_v_*)*^T^*. Note that the endpoints initialization is same as the procedure presented by Smith *et al*. [8].

Besides the location of line’s endpoints used for measurement model, we also considered several additional parameters: the position of projected intersection point *p_ip_* which has been calculated via measurement model and the line descriptor [*ρ*, *θ*]*^T^* in Hough space. They are applied as the auxiliary parameters for our proposed data association strategies, and we will concentrate on these topics in the following sections. A step-by-step procedure of the complete edge/line extraction from the camera is as follows:

Step 1: Pre-process the acquired image to filter out different noise signals;
Step 2: Select the region of interest (ROI);
Step 3: In the ROI, detect the horizontal edges by the Sobel operator combined with thresholding;
Step 4: *shrink* and *clean* morphological operations on all edges to eliminate those corresponding to dynamic objects;
Step 5: Remove the edges whose length is less than the length threshold;
Step 6: Implement *thicken* operation to recover the interested edges;
Step 7: Calculate the pixel coordinates of line endpoints and projected intersection point, and descriptors in Hough space.

### 3.2. Data Association Based on Homography Transformation Matrix

**Figure 3.**
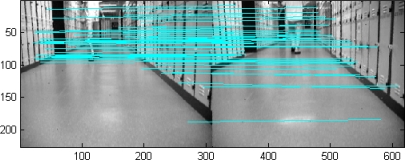
Matched point determined by SIFT descriptors.

Sampling is considered very important in Nearest Neighbor data association methods. In the reference works [1–5,8], samples in a window region are used to match the predicted features and calculate the innovation. However, the computation pixel by pixel in the predefined region is a little bit repetitious. In this subsection, we suggest a homography transformation matrix based data association (HTMDA) method. This matrix is estimated by the matched points between two images with the help of SIFT descriptors. HTMDA firstly applies the SIFT mechanism to detect the matched points between any two images. Because of advantages of the SIFT, as is shown in Figure 3 the matched points are obviously unsusceptible to the moving object (the person here). Therefore it is reasonable to apply them as the stable points to determine the homography transformation matrix. By these matched points the homography transformation matrix **M** and its covariance **Σ_M_** can be estimated using the computational procedure of MLE technique [10].

After the estimations of **M** and **Σ_M_** are obtained, the predicted pixel coordinates *p̂^l^* of line endpoints and projected intersection point in the image plane can be expressed as:

(5) $${\hat{p}}^{l}=Mp^{m}$$

where *p^m^* is the coordinates of the line endpoints and projected intersection point stored in the map (note that projected intersection point is not the component of the state variable). The coordinates of the observed feature in the image is marked as *p^l^*, and the definition of Mahalanobis distance is:

(6) $$s_{m}={\left(p^{l}-{\hat{p}}^{l}\right)}^{T}\Sigma_{M}^{-1}\left(p^{l}-{\hat{p}}^{l}\right)$$

When estimating **M** and **Σ_M_**, we have considered the pixel error in both images as well as the propagation in Equation (5). The covariance in observation and prediction can be regarded as being led by the covariance **Σ_M_**. That is why here we use **Σ_M_** for the Mahalanobis distance computation. As many popular data association algorithms, Mahalanobis distance can be treated as the criteria for data association. Hence in this work, if two values of Mahalonbis distance meet the following condition:

(7) $$s_{\mathrm{mi}}\leq \chi^{2}(\alpha ,n),i=1,2$$

(these two Mahalanobis distance values are calculated by any two points, for example two endpoints or one endpoint and one projected intersection point, located on the observed line), then the observed line can be associated with the line stored in the map, labelled as 1, otherwise it is a new feature, marked as 0. Where *α* is the statistically significant level *i.e.*, P-value and *n* is the number of degrees of freedom.

Looking back through the implemented process of the proposed HTMDA, compared with the related work in Section 2, instead of directly applying SIFT descriptors as the natural features for data association, we emphasize using the SIFT mechanism to determine the matched points between any two images and then apply these matched points to estimate the homography transformation matrix and its related covariance. The data association is based on this estimated matrix and its covariance. Additionally the main difference between our defined Mahalanobis distance in Equation (6) and the formula in Gil’s work [26] is that the distance is constructed only by **Σ_M_** and the pixel coordinates without any SIFT descriptor.

#### 3.2.1. Practical Considerations on Data Association

Sometimes the position of the predicted image line endpoints may be outside the image range, and we cannot use the criteria (7) above to determine associated features. For this special case, we employ the Hough space parameters presented in previous subsection to handle the data association problem, and adopt an alternative criterion, that is, to test whether the predicted endpoints lie on the observed image lines. The ends lie on the lines if and only if ${\left({\hat{p}}_{h}^{l}\right)}^{T}l^{m}=0$. We relax this condition practically as:

(8) $${\left({\hat{p}}_{h}^{l}\right)}^{T}l^{m}<\varepsilon$$

where *ε* is an arbitrarily small positive quantity, *l^m^* = (cos*θ*, sin*θ*, −*ρ*)*^T^* is the homogeneous representation for observed image lines by the Hough space parameters, and ${\hat{p}}_{h}^{e}={\left({\hat{x}}_{u},{\hat{y}}_{v},1\right)}^{T}$ is the homogenous pixel coordinates of predicted line endpoints. If there exists two predicted line endpoints that meet the condition:

(8-1) $${\left({\hat{p}}_{\mathrm{hi}}^{l}\right)}^{T}l^{m}<\varepsilon ,i=1,2$$

or one predicted line endpoint/projected-intersection-point meets condition (7) and another predicted line endpoint makes the criterion (8) true, then the observed line is matched with the line feature stored in the map or else it is a new one.

It is impractical and time consuming to compute all **M** and **Σ_M_** between the most recent image and all previous ones. In this study we captured an image per 2 s and calculated **M**, **Σ_M_** by using the newest grabbed image and the two latest ones with the sliding window technique, because the robot moves in an intermediate speed and after about 4 s some features stored in the map could probably disappear in current image. Algorithm 1 illustrates our HTMDA algorithm.

**Algorithm 1.** HTMDA algorithm.

**HTMDA Algorithm**
// Input: observed lines parameters, the 3 most recent images
//Output: data association matrix DA
[desCur, locCur ] = sift(CurrentImg); // Find SIFT keypoints for current captured image. The outputs are
// desCur: descriptor for the keypoint; locCur: keypoint location
**for** each observed line i
**for** k = 2:−1:1
[desK, locK] = sift(Img(k)); // Find SIFT keypoints for kth image.
// Estimating **M** and **Σ_M_**
[M(k), Σ_M_(k)] = HomographyEstimation(locCur, locK, *σ*_C_); // *σ*_C_ is the variance of image noise
// Observation prediction
**for** each line feature j stored in map
EndsPred (j) = M(k)EndsMap(j); // Equation (5)
s_m_ = (EndsObs(i) – EndsPred(j)) · (Σ_M_(k))^−1^ · (EndsObs(i) − EndsPred(j))*^T^*; // Equation (6)
**if** (condition (7) is true) // Any two Mahalanobis distance values satisfy the condition (7)
DA(i, j, k) = 1;
**else if** ((condition (8-1) is true) || (condition (7) && condition (8) are true))
// Two predicted line points locate on the same line, or one Mahalanobis distance value meets
// condition (7) and one predicted line point lies on the observed line.
DA(i, j, k) = 1;
**else**
DA(i, j, k) = 0;
**end if**
**end**
**if** ∼isZero(DA(i, :, :))
**continue;**
**end if**
**end**
**end**

## 4. Sensor Fusion Strategy

As was mentioned in Section 1, this study is a natural extension of our prior research [12]. In that work, we proposed a robust regression model by MM-estimate for the segment based SLAM in dynamic environments. The segments (named laser segments) were extracted from the raw laser rangefinder data and most of the outliers related to moving objects were eliminated. However, if these dynamic objects momentary start and stop several times, they could probably be treated as segment features by using a robust regression model and be misincorporated into the state variables, which will deteriorate the performance of SLAM. Since the lines extracted from the monocular camera are almost static after necessary processing stated in Subsection 3.1, we combine these image line features with laser segments and apply Bayesian decision as the feature fusion strategy to remove those pseudo segments reflected in the laser segments. Furthermore, we suggest a modified MPEF-SLAM to incorporate the state estimates obtained from the individual monocular and laser SLAM. With this modified MPEF-SLAM, the covariance of the robot pose is reduced so that the accuracy of the localization can be improved.

### 4.1. Line Feature Fusion

The purpose of the feature fusion is to remove the pseudo laser segments corresponding to dynamic objects. Before the implementation of feature fusion, it is necessary to figure out the laser segments and image lines in the same sensor detection range. As the horizontal field of view (HFOV) of the monocular camera has a limited visual angle, it is feasible to extract the laser segments within this HFOV. That is, when a frame of raw laser data is received, those located outside the HFOV are filtered out. For these filtered raw data, the robust regression model [12] is employed to extract the laser segments and estimate the segments parameters. These laser segments are defined as the laser segment set labeled as *SF_L_*. Similarly, we can obtain the image lines and compute their parameters from the grabbed image, as well define the image line set as *SF_I_*. The pre-processing procedure above ensures that these two sets of line features are extracted within the same detection range. After that, the Bayesian decision fusion rule [30] is applied to determine the matched features via exhaustive algorithm in these two feature sets. The fusion rule is:

$$\frac{p\left(y|H_{1}\right)}{p\left(y|H_{0}\right)}\overset{H_{1}}{\underset{H_{0}}{≷}}\frac{p\left(H_{1}\right)\left(C_{10}-C_{00}\right)}{p\left(H_{0}\right)\left(C_{01}-C_{11}\right)}:=\mathrm{LR}(y)\overset{H_{1}}{\underset{H_{0}}{≷}}\eta$$

where *p*(**y**|**H***_i_*) *i* = 0,1 is the conditional probability of event **y** when the hypothesis **H***_i_* is true. Event **y** means the feature matching. In this study we define event **y** = [**z***_C_*, **z***_L_*]*^T^*, **z***_C_* is the image lines parameters for feature fusion and **z***_L_* is of the laser segments. Null hypothesis **H**_0_ means the laser segments are assumed to assign to the noise, and relevant alternative hypothesis **H**_1_ implies the laser segments probably are related to image line features. *p*(**H***_i_*) is the probability when hypothesis **H***_i_* is true. *C_ij_*, *i* = 0,1, *j* = 0,1, represents the cost of declaring **H***_i_* true when **H***_j_* is actually true. For **H**_0_, we choose event **y** as [**z***_C_*, **z***_L_*]*^T^* = [*s_C_* + *v_C_*, *v_L_*]*^T^*, *s_C_* is the parameters of image lines with the zero mean and covariance ***R****_C_*, *v_C_* and *v_L_* which have zero mean and covariance $\sigma_{C}^{2}$ and $\sigma_{L}^{2}$ are mutually independent additional sensor noise of the camera and laser. Event **y** for testing **H**_1_ is [**z***_C_*,**z***_L_*]*^T^* = [*s_C_* + *v_C_*, *s_L_* + *v_L_*]*^T^* where *s_L_* is the laser segment parameters with the zero mean and covariance ***R****_L_*. Noted that the parameter *s_C_* of image lines is [*h_k_*_,_ *ϕ_k_*]*^T^*. As for the calculation of *s_L_* and *R_L_*, the interested reader may refer to our prior work [12] for more detail. Generally, the monotonically increasing natural logarithm rule is considered, that is:

(9) $$\text{ln}\mathrm{LR}(y)\overset{H_{1}}{\underset{H_{0}}{≷}}\text{ln}\eta$$

Suppose that *p*(**H**_0_) = *p*(**H**_1_) = 0.5, *C*_01_ = *C*_10_ =1 and *C*_00_ = *C*_11_ = 0, which means that the cost for mistaken decision is much more than that for correct decision, then *p*(**y**|**H**_0_) ∼ N(**0**,**Σ**_0_) and *p*(**y**|**H**_1_) ∼ N(**0**,**Σ**_1_). Here:

(10) $$\Sigma_{0}=\left(\begin{array}{cc} R_{C}+{I\sigma }_{C}^{2} & 0\\ 0 & \sigma_{L}^{2} \end{array}\right),\Sigma_{1}=\left(\begin{array}{cc} R_{C}+{I\sigma }_{C}^{2} & 0\\ 0 & R_{L}+I\sigma_{L}^{2} \end{array}\right),I\;\text{is the identity matrix}$$

and the decision rule (9) is equal to:

(11) $$-\frac{1}{2}\left({\left[\begin{array}{c} z_{C}\\ z_{L} \end{array}\right]}^{T}\sum_{1}^{-1}\left[\begin{array}{c} z_{C}\\ z_{L} \end{array}\right]-{\left[\begin{array}{c} z_{C}\\ z_{L} \end{array}\right]}^{T}\sum_{0}^{-1}\left[\begin{array}{c} z_{C}\\ z_{L} \end{array}\right]\right)\overset{H_{1}}{\underset{H_{0}}{≷}}\text{ln}\frac{\left|\sum_{1}\right|}{\left|\sum_{0}\right|}$$

With rule (11), we validate all the laser segments located in HFOV. If **H**_0_ is accepted then the laser segment is the outlier, otherwise if **H**_1_ is accepted then it is the definite static feature. Actually, employing the exhaustive algorithm to search matched features in two line sets is a tedious work. To handle this problem, for two endpoints of each image line we can respectively compute approximate angles from robot head (*i.e.*, the *x*-axis of robot frame) via parameter [*h_k_*, *ϕ_k_*]*^T^* as well as determine the angular interval [γ*_C_*_1_, γ*_C_*_2_]. Similarly, when extracting laser segments, according to the laser scanning resolution it is easy to obtain the angles from robot head for two endpoints of each segment and the related angle ranges [γ*_L_*_1_, γ*_L_*_2_]. If these two groups of angle boundaries are close, we use Equation (11) to check whether the laser segments are outlier or not. By this technique, the search work can be reduced.

### 4.2. Modified MPEF-SLAM

In [30], a framework of MPEF for a Kalman filter was proposed. It led to a lower covariance for fused state estimates compared with each individual one, as well maintaining the optimal estimation. We extended the idea of MPEF in this paper to EKF SLAM problem, and developed a modified MPEF-SLAM framework. It incorporates two individual parallel-running EKF SLAM processes: monocular SLAM and laser SLAM to build a fused EKF SLAM procedure.

**Algorithm 2.** Modified MPEF-SLAM algorithm.

**Fusion SLAM based on Modified MPEF Algorithm**
// Robot pose initialization
[**x**^1^_v0_, P^1^_v0_] = PoseInitialization(Camera); // Initialization of monocular SLAM, **x**_v_ and **P**_v_ are the initial values
*σ*_C_ = getSensorError(Camera);
[**x**^2^_v0_, P^2^_v0_] = PoseInitialization(Laser); // Initialization of laser SLAM
[*σ*_range_, *σ*_bearing_] = getSensorError(Laser); // Obtaining the noise parameters of laser sensor
Q = createQ(*σ*_tra_, *σ*_rot_); // Obtaining the noise parameters of intrinsic sensor, *i.e.*, encoder
// Line Feature initialization
SegC = HorizontalEdge(image); // Horizontal line extraction from 1^st^ captured image
[**z**^1^_0_, P^1^**_z_**_0_, R_C_] = intializeNewFeature(SegC, camPar, **x**^1^_v0_, P^1^_v0_, *σ*_C_); // Image line feature initialization. camPar:
// intrinsic parameters of the camera
SegL = LineExtraction(laserdata); // Segment extraction from 1^st^ frame of laser data
[**z**^2^_0_, P^2^**_z_**_0_, R_L_] = intializeNewFeature(SegL, **x**^2^_v0_, P^2^_v0_, *σ*_range_, *σ*_bearing_); // Segment feature initialization
// State variable and related covariance initialization
**X**^1^_0_ = createX(**x**^1^_v0_, **z**^1^_0_); P^1^_0_ = cerateP(P^1^_v0_, P^1^**_z_**_0_); // For monocular SLAM
**X**^2^_0_ = createX(**x**^2^_v0_, **z**^2^_0_); P^2^_0_ = cerateP(P^2^_v0_, P^2^**_z_**_0_); // For laser SLAM
// Fused robot pose initialization $x_{v0}^{f}=x_{v0}^{2};P_{0}^{f}=P_{v0}^{2}$;
// Main loop
k = 1;
**while** isRobotRunning()
u_k_ = getControl(k); // Obtaining control variables
[**X^1^**_k|k_, P^1^_k|k_, **X^1^**_k|k-1_, P^1^_k|k-1_ ] = MonoSLAM(**X^1^**_k-1_, P^1^_k-1_, u_k_, Q, R_C_, getObservation(image_k_));
[**X^2^**_k|k_, P^2^_k|k_, **X^2^**_k|k-1_, P^2^_k|k-1_ ] = LaserSLAM(**X^2^**_k-1_, P^2^_k-1_, u_k_, Q, R_L_, getObservation(laser_k_));
// Do MPEF procedure
$\begin{array}{ll} x_{v(k|k)}^{f} & =x_{v(k|k)}^{f}+P_{k|k}^{f}\sum_{i=1}^{2}{\left(P_{k|k}^{i}\right)}^{-1}\left(x_{k|k}^{i}-x_{k|k-1}^{i}\right)\\ & =W{\left[x_{v(k|k)}^{f},x_{k|k}^{1},x_{k|k-1}^{1},x_{k|k}^{2}-x_{k|k-1}^{2}\right]}^{T} \end{array}$; ${\left(P_{k|k}^{f}\right)}^{-1}-{\left(P_{k|k-1}^{f}\right)}^{-1}=\sum_{i=1}^{2}{\left(P_{k|k}^{i}\right)}^{-1}-{\left(P_{k|k-1}^{i}\right)}^{-1})$;
**// W** is the weight matrix, and $W=\left[I,P_{k|k}^{f}\;{\left(P_{k|k}^{1}\right)}^{-1},-P_{k|k}^{f}\;{\left(P_{k|k}^{1}\right)}^{-1},P_{k|k}^{f}\;{\left(P_{k|k}^{2}\right)}^{-1},-P_{k|k}^{f}\;{\left(P_{k|k}^{2}\right)}^{-1}\right]$
// Propagate backward the MPEF results to each individual SLAM
$x_{v(k|k-1)}^{i}=x_{v(k|k-1)}^{f};P_{k|k-1}^{i}=P_{k|k-1}^{f};//i=1,2$
// Update individual covariance
$P_{k|k}^{i}=\text{update}\left(P_{k|k-1}^{i},P_{k|k-1}^{f},P_{k|k-1}^{i\_b}\right);//P_{k|k}^{b}=P_{k|k}^{f}\leq P_{k|k}^{i},i=1,2$
$P_{k|k-1}^{i\_b}=P_{k|k}^{i}$;
k = k + 1;
**end**

In the modified MPEF-SLAM framework, the state variable and its covariance in each individual SLAM are first fused by a fusion-weighted matrix to obtain a fused state variable and covariance. After that, the fused state variable and covariance are propagated backward to each individual SLAM for updating the individual state variable and related covariance. By this updating scheme, the covariance matrices of the fused and individual state variable are decreased, even though the fused estimation could not be kept at an optimal value. The details on the theoretical derivation of the modified MPEF-SLAM are described in Appendix. The purpose of this modified MPEF-SLAM is to improve the accuracy of localization. We sketched our fusion SLAM algorithm in Algorithm 2. The superscript *i* indicates the type of the sensor, 1 for monocular camera and 2 for laser; *f* means fusion and *b* stands for back propagation.

## 5. Experimental Results

We conducted extensive experiments in the corridor just outside the control laboratory of the Electrical Engineering Department. The mobile robot platform used for experimental studies was the Pioneer 2DX mounted with a Canon VCC4 monocular camera, a SICK LMS200 laser rangefinder and a 16-sonar array. The camera was calibrated by the Calibration Toolbox (available online: http://www.vision.caltech.edu/bouguetj/calib_doc/) and the intrinsic parameters are listed in Table 1. A sequence of images as well a frame of laser data were collected when the mobile robot was moving with an average speed of 300 mm/s using ARIA and the OpenCV class library. The sampling time *T_s_* for feature extraction and control values acquisition is 2 s. There were several people walking through the corridor at normal speed around the robot. Sometimes they slowed down or stopped completely at some place. After obtaining sensor data, we implemented the SLAM and sensor fusion offline in MATLAB environment on a desktop PC with Pentium 4 3.0 GHz CPU and 1G RAM. The experiments were designed to validate our sensor fusion strategy and data association algorithm.

### 5.1. Testing the Feature Fusion Strategy

**Figure 4.**
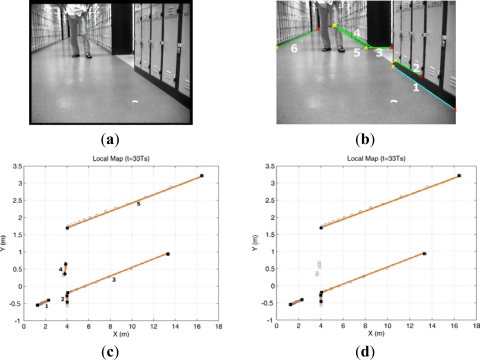
Local mapping result at the 33rd sample time. (**a**) The original captured image. A person stood in front of the robot for a moment. (**b**) The extracted image lines and their endpoints in ROI. The cyan line is the first extracted one and numbered as 1. (**c**) The extracted laser segments within the HFOV and a pseudo segment (segment 4) related to standing person. (**d**) After integrating the lines information extracted from images, the incorrect segment was removed.

In this experiment, a person stood in front of the robot for few minutes shown in Figure 4(a). The size of ROI defined in Figure 2(a) is *u*: [0,320] pixel and *v*: [40,240] pixel. The extracted image lines with the endpoints in this ROI are illustrated in Figure 4(b). Also the segments obtained from laser sensor are displayed in Figure 4(c), and it can be seen that several laser segments, for example laser segment 4, are pseudo features which are related to the dynamic objects. It is clear that these pseudo laser segments can not be removed by the MM-estimate based method proposed in our previous work. To delete these pseudo features, by using the line feature fusion strategy describe in Section 4, we incorporated the image lines with the laser segments and tested all possible hypothesis to determine which laser segment is not the feature.

Table 2 lists the hypothesis test results. It can be seen that laser segment 4 did not match any image line and it can be eliminated from the set *SF_L_*.

**Figure 5.**
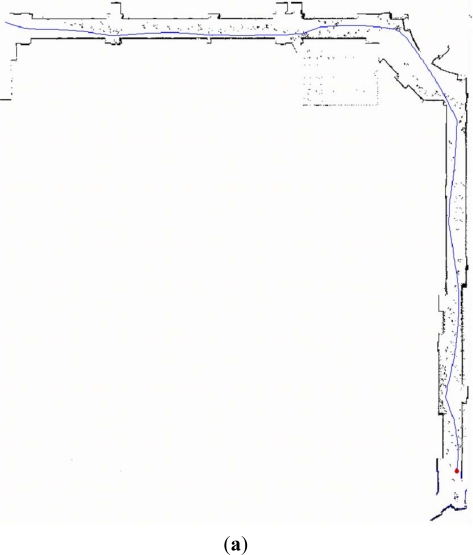

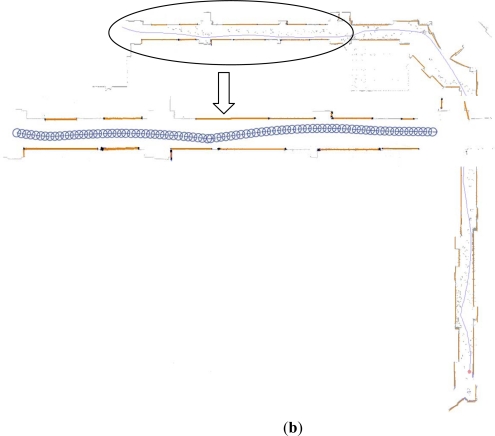
The laser SLAM results with the feature fusion. (**a**) The robot trajectory and the grid map plotted by the software of ActivMedia Co. using raw laser data; (**b**) The final built map using feature fusion where the part circled by the ellipse is enlarged to show the details. orange: the segment map after fusion; light gray: the grid map for comparison.

Additionally, it can be found that laser segment 3 correlated with image lines 4 and 5. This is because laser segment 3 concurrently located in the angle interval determined by image lines 4 and 5 respectively. However, it only related to line 5 according to the fusion rule. We applied this feature fusion strategy in the whole EKF laser SLAM process and the result after feature fusion is shown in Figure 5.

Figure 5(a) is the robot trajectory and grid map plotted by the software of ActivMedia Company with raw laser data. It is obvious that parts of grid map are contaminated by the walking persons. Those raw laser data corresponding to the moving objects lead to the extraction of pseudo laser segments. Fortunately, with the proposed feature fusion method they are almost removed, which is shown in Figure 5(b). And the grid map is overlaid in light gray color for comparison. Furthermore, it can be found in Figure 5(b) that a few of raw laser data related to the static segment features are lost in the final map.

The reasons for this case are: one is the locations of these laser data are out of the HFOV of the camera, the other is the assumptions of proposed Bayesian fusion rule is strict (*i.e.*, pessimistic condition) so that a segment related to the real static object is mis-deleted as the pseudo one. With this experiment, we can state that the feature fusion method is competent for disposing pseudo and confused features.

### 5.2. Testing the Modified MPEF-SLAM

**Figure 6.**
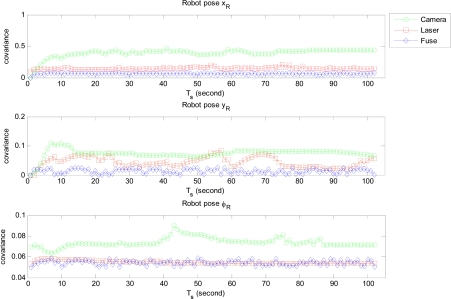
The estimated covariance of fused and individual robot pose. red line: estimated covariance in laser SLAM; green line: estimated covariance in monocular SLAM; blue lines: estimated covariance in MPEF-SLAM.

We firstly ran two individual EKF SLAM: monocular SLAM and laser SLAM procedures in parallel mode. The state variable and its covariance obtained respectively from each individual SLAM were integrated to compute the fused state variable and related covariance of the MPEF-SLAM. Finally the fused state variable and covariance were propagated back to monocular and laser SLAM respectively for updating the individual state variables to improve the localization accuracy. Figure 6 illustrates the covariance of the fused and individual robot pose. It can be seen that the covariance of the position: *x_R_* and *y_R_* is obviously reduced after fusion. However, the covariance of the orientation *ϕ_R_* is similar to the value from laser SLAM, but it is more efficient than that from the monocular SLAM. This is because the covariance of the state variable in laser SLAM contributes more for computing the weighted matrix.

**Figure 7.**
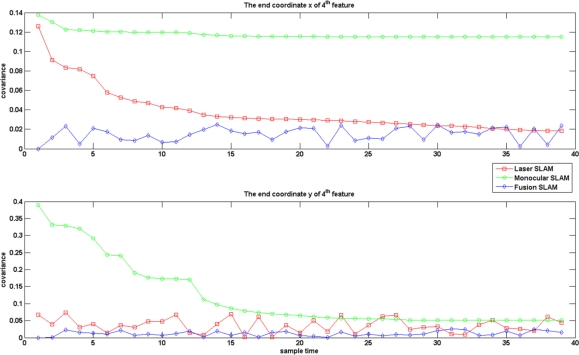
The estimated covariance of an endpoint of one line feature. Red line: estimated covariance in laser SLAM; Green line: estimated covariance in monocular SLAM; Blue lines: estimated covariance in MPEF-SLAM.

Figure 7 gives the results of covariance on an endpoint of one line feature. We note that the selected line features displayed in Figure 7 for validation are the ones always appearing in 40 consistent images during the experiment. It seems that the covariance of the line endpoints after sensor fusion is also reduced. With these experiments, we may state that the MPEF-SLAM decreases the covariance of state variables and increase the accuracy of localization.

### 5.3. Testing the HTMDA

**Figure 8.**
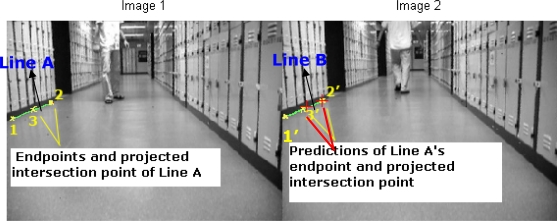
The endpoints and projected intersection point of the lines in the stored and captured images. The image 1 (on the left) is captured at the 57th sample time and the right one (image 2) is at the 58th sample time.

In the defined ROI, we compute **M** and **Σ_M_** via the current captured image (labeled as image 2 in Figure 8) and one image stored in image sequence buffer, for example the previous image (labeled as image 1 in Figure 8). After that with the estimated **M** and **Σ_M_** we selected one pair of lines to demonstrate our HTMDA method. As shown in Figure 8, we marked endpoints as 1, 2 and projected intersection point as 3 for Line A of image 1. Those for Line B observed in image 2 are as 1′, 2′ and 3′. According to Equation (5), we obtained the predictions of 2′ and 3′ stressed in red cross in image 2. It can be seen that the prediction of 2′ almost coincides with the 2′, and the prediction of 3′ locates at Line B but is a little bit far from 3′. The position of the prediction of endpoint 1′ is out of the bound of image 2. In this case, we may use condition (7) to decide if Line B is associated with Line A or not, but it is false. Hence the condition in Equation (8-1) has to be considered to test whether the predictions of 1′ and 3′ lie on Line B. Obviously this condition is true. Therefore, we can determine that the observed Line B in the captured image is matched with the Line A stored in the map.

**Figure 9.**
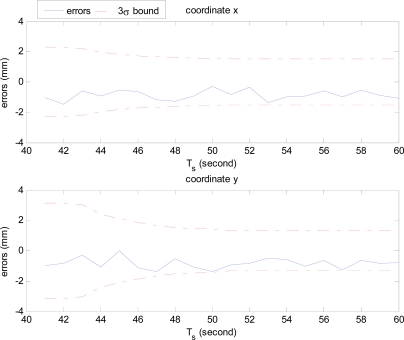
Errors between the actual and estimated location of endpoint 2 from 40th to 60th sample time. The 99% confidence limit is shown in red dash-dot *line.*

Figure 9 shows the errors of HTMDA for the known endpoint 2 of Line A in Figure 8. Because there is no device in our present experimental conditions for detecting the ground truth of the features, we provisionally measured the truth value by hand as accurate as possible, which follows the process of [31]. Line A appears in around 20 sequential images. As displayed in Figure 9, the actual feature estimation errors are bounded within the 3*σ* limits, which demonstrates the effectiveness and consistency of the proposed HTMDA.

## 6. Conclusions

In this paper, we suggest a sensor fusion strategy including feature fusion and modified MPEF-SLAM modules for the SLAM task of autonomous mobile robots in dynamic environments. Our feature fusion policy incorporates the line features extracted by a monocular camera with the segments represented by robust regression model from a laser sensor, the purpose of which is to remove the potential pseudo laser segments corresponding to the moving objects. The modified MPEF-SLAM combines state variable estimates obtained from individual SLAM procedure (monocular and laser SLAM), and respectively propagates the fused state variable backward to each SLAM process to reduce the covariance of the state variable of individual SLAM furthermore improve the accuracy of localization. Additionally, for the data association problem in monocular SLAM we present a new method based on homography transformation matrix. It relaxes redundant computational procedures compared with the algorithm based on pixel by pixel computation. Experimental results verify the performance of the proposed sensor fusion strategy and data association algorithm. The planned future work will include improvement of the feature fusion module on how to use the laser data located outside the HFOV and extension of sensor fusion modules such as sensor management, active sensor. Another promising direction is on developing an online implementation for the proposed HTMDA and MPEF-SLAM algorithm by embedded hardware and technique.

## Figures and Tables

**Table 1.** Intrinsic Parameters.

**Item**	**Value**
Focal length	fc = [365.12674 365.02905]
Principal point	cc = [145.79917 114.50956]
Skew factor	alpha_c = 0.000
Distortion factor	kc = [−0.22776, 0.36413, −0.00545, −0.00192, 0.000]
Pixel std	err = [0.10083 0.10936]

**Table 2.** The hypothesis test of feature fusion.

**Number of Segments from laser**	**Number of lines in image**					
	**1**	**2**	**3**	**4**	**5**	**6**
1	**H**_1_	×	×	×	×	×
2	×	×	**H**_1_	×	×	×
3	×	×	×	**H**_0_	**H**_1_	×
4	×	×	×	×	×	×
5	×	×	×	×	×	**H**_1_

× means no fusion process is implemented. **H**_0_ means the laser segments are assumed to assign to the noise, and **H**_1_ implies the laser segments probably are related to image line features.

## Appendix

The motion models for two SLAM procedures are identical and represented as:

(12) $$x_{v(k+1)}^{i}=f\left(x_{v(k)}^{i},u_{k}\right)+v_{k};i=1,2;k=0,1,...$$

and the observation model is:

(13) $$z_{k}^{i}=h^{i}\left(x_{v(k)}^{i}\right)+w_{k}^{i};i=1,2;k=0,1,...$$

where the motion noise *v_k_* and measurement noise $w_{k}^{i}$ are both zero mean random variables independent of each other and are not cross correlated. Their covariance matrices are *Q_k_* and $R_{k}^{i}$ respectively. *i* = 1,2 has the same meaning as stated in Section 4.2. To compare performances between fused and distributed filtering, the stacked measurement equation is:

(14) $$z_{k}=h\left(x_{v(k)}^{i}\right)+w_{k}^{};i=1,2;k=0,1,...$$

where:

$$z_{k}={\left[{\left(z_{k}^{1}\right)}^{T},{\left(z_{k}^{2}\right)}^{T}\right]}^{T},h\left(x_{v(k)}^{i}\right)=\left[\begin{array}{l} h^{1}\left(x_{v(k)}^{1}\right)\\ h^{2}\left(x_{v(k)}^{2}\right) \end{array}\right],w_{k}={\left[{\left(w_{k}^{1}\right)}^{T},{\left(w_{k}^{2}\right)}^{T}\right]}^{T}$$

and the covariance of the noise *w*_k_ is given by:

$$\mathrm{Cov}\left(w_{k}\right)=\mathrm{diag}\left(R_{k}^{1},R_{k}^{1}\right)=R_{k}$$

Each individual EKF SLAM is:

(15) $$x_{v(k|k)}^{i}=x_{v(k|k-1)}^{i}+K_{k}^{i}\left[z_{k}^{i}-h^{i}\left(x_{v(k|k-1)}^{i}\right)\right]=x_{v(k|k-1)}^{i}-K_{k}^{i}h^{i}\left(x_{v(k|k-1)}^{i}\right)+K_{k}^{i}z_{k}^{i}$$

(16) $$K_{k}^{i}=P_{k|k-1}^{i}{\left(H_{k}^{i}\right)}^{T}{\left(S_{k}^{i}\right)}^{-1},S_{k}^{i}=H_{k}^{i}P_{k|k-1}^{i}{\left(H_{k}^{i}\right)}^{T}+R_{k}^{i}$$

(17) $$P_{k|k}^{i}=\left(I-K_{k}^{i}H_{k}^{i}\right)P_{k|k-1}^{i},P_{k|k-1}^{i}=F_{k}P_{k-1|k-1}^{i}F_{k}^{T}+Q_{k}$$

where *F*_k_ and $H_{k}^{i}$are the Jacobian matrix with respect to the state variable **x***_vk_*. From Equations (16,17), we have:

(18) $$K_{k}^{i}=P_{k|k}^{i}{\left(H_{k}^{i}\right)}^{T}{\left(R_{k}^{i}\right)}^{-1}$$

(19) $${\left(P_{k|k}^{i}\right)}^{-1}={\left(P_{k|k-1}^{i}\right)}^{-1}+{\left(H_{k}^{i}\right)}^{T}{\left(R_{k}^{i}\right)}^{-1}H_{k}^{i}$$

For the fused EKF SLAM, there are similar formulas:

(20) $$x_{v(k|k)}^{f}=x_{v(k|k-1)}^{f}+K_{k}^{f}\left[z_{k}-h\left(x_{v(k|k-1)}^{f}\right)\right]=x_{v(k|k-1)}^{f}-K_{k}^{f}h\left(x_{v(k|k-1)}^{f}\right)+K_{k}^{f}z_{k}$$

(21) $$K_{k}^{f}=P_{k|k}^{f}H_{k}^{T}R_{k}^{-1}$$

where $H_{k}={\left[{\left(H_{k}^{1}\right)}^{T},{\left(H_{k}^{2}\right)}^{T}\right]}^{T}$. Also the covariance of the fused state variable is deduced as:

(22) $${\left(P_{k|k}^{f}\right)}^{-1}={\left(P_{k|k-1}^{f}\right)}^{-1}+H_{k}^{T}R_{k}^{-1}H_{k}={\left(P_{k|k-1}^{f}\right)}^{-1}+\sum_{i=1}^{2}{\left(H_{k}^{i}\right)}^{T}{\left(R_{k}^{i}\right)}^{-1}H_{k}^{i}$$

According to Equations (19) and (22), $P_{k|k}^{f}$ can be represented by $P_{k|k}^{i}$, *i.e.*,

(23) $${\left(P_{k|k}^{f}\right)}^{-1}-{\left(P_{k|k-1}^{f}\right)}^{-1}=\sum_{i=1}^{2}{\left(H_{k}^{i}\right)}^{T}{\left(R_{k}^{i}\right)}^{-1}H_{k}^{i}=\sum_{i=1}^{2}\left[{\left(P_{k|k}^{i}\right)}^{-1}-{\left(P_{k|k-1}^{i}\right)}^{-1}\right]$$

Multiplying **z***_k_* at both side of Equation (21), we have:

(24) $$K_{k}^{f}z_{k}=P_{k|k}^{f}H_{k}^{T}R_{k}^{-1}z_{k}=P_{k|k}^{f}\sum_{i=1}^{2}{\left(H_{k}^{i}\right)}^{T}{\left(R_{k}^{i}\right)}^{-1}z_{k}$$

and by Equation (15) we obtain:

(25) $$\begin{array}{l} K_{k}^{i}z_{k}^{i}=x_{v(k|k)}^{i}-\left[x_{v(k|k-1)}^{i}-K_{k}^{i}h^{i}\left(x_{v(k|k-1)}^{i}\right)\right]\Rightarrow \\ P_{k|k}^{i}{\left(H_{k}^{i}\right)}^{T}{\left(R_{k}^{i}\right)}^{-1}z_{k}^{i}=x_{v(k|k)}^{i}-\left[x_{v(k|k-1)}^{i}-K_{k}^{i}h^{i}\left(x_{v(k|k-1)}^{i}\right)\right]\Rightarrow \\ {\left(H_{k}^{i}\right)}^{T}{\left(R_{k}^{i}\right)}^{-1}z_{k}^{i}={\left(P_{k|k}^{i}\right)}^{-1}\left(x_{v(k|k)}^{i}-\left[x_{v(k|k-1)}^{i}-K_{k}^{i}h^{i}\left(x_{v(k|k-1)}^{i}\right)\right]\right) \end{array}$$

Substituting Equation (25) into Equation (24), and then substituting Equations (21) and (24) into Equation (20), we find:

(26) $${\left(P_{k|k}^{f}\right)}^{-1}x_{v(k|k)}^{f}={\left(P_{k|k}^{f}\right)}^{-1}x_{v(k|k-1)}^{f}-H_{k}^{T}R_{k}^{-1}h\left(x_{v(k|k-1)}^{f}\right)+\sum_{i=1}^{2}{\left(P_{k|k}^{i}\right)}^{-1}\left(x_{v(k|k)}^{i}-\left[x_{v(k|k-1)}^{i}-K_{k}^{i}h^{i}\left(x_{v(k|k-1)}^{i}\right)\right]\right)$$

Actually in each iteration, $h\left(x_{v(k|k-1)}^{f}\right)$ and $h^{i}\left(x_{v\left(k|k-1\right)}^{i}\right)$ are constant matrix calculated by the values of $x_{v(k|k-1)}^{f}$and $x_{v(k|k-1)}^{i}$. Therefore $H_{k}^{T}R_{k}^{-1}h\left(x_{v(k|k-1)}^{f}\right)$ and ${\left(H_{k}^{i}\right)}^{T}{\left(R_{k}^{i}\right)}^{-1}h^{i}\left(x_{v(k|k-1)}^{i}\right)$ are linear items. With the property of linearity, we may determine:

(27) $$H_{k}^{T}R_{k}^{-1}h\left(x_{v(k|k-1)}^{f}\right)=\sum_{i=1}^{2}{\left(H_{k}^{i}\right)}^{T}{\left(R_{k}^{i}\right)}^{-1}h^{i}\left(x_{v(k|k-1)}^{i}\right)$$

By Equations (18) and (27), Equation (26) is rewritten as:

(28) $$x_{v(k|k)}^{f}=x_{v(k|k-1)}^{f}+P_{k|k}^{f}\sum_{i=1}^{2}{\left(P_{k|k}^{i}\right)}^{-1}\left(x_{v(k|k)}^{i}-x_{v(k|k-1)}^{i}\right)$$

It is necessary to note that Equations (23) and (28) manifest the relationship of the state variables as well as the covariance matrix in the fused and individual EKF SLAM. From Equation (28), the weight matrix for each individual state variable can be determined. That is:

(29) $$W=\left[I,P_{k|k}^{f}{\left(P_{k|k}^{1}\right)}^{-1},-P_{k|k}^{f}{\left(P_{k|k}^{1}\right)}^{-1},P_{k|k}^{f}{\left(P_{k|k}^{2}\right)}^{-1},-P_{k|k}^{f}{\left(P_{k|k}^{2}\right)}^{-1}\right]$$

If the latest fused state estimate $x_{v(k|k)}^{f}$ is broadcasted to every individual state estimate as the back propagation (named feedback), we can prove that the covariance of state variable is reduced with this feedback but the performance of the fused EKF SLAM is unchanged with and without the feedback.

To maintain the identity with [30], we apply the same symbols and assumptions, and the general symbols **x̂** and *P̂* mean the state variable and its covariance when the feedback is considered. Concerning the feedback, the individual and fused one-step predictions are:

(30) $$x_{v(k|k-1)}^{i}=f\left({\hat{x}}_{v(k-1|k-1)},u_{k-1}\right),{\hat{P}}_{k|k-1}^{i}={\hat{P}}_{k|k-1},\;i=1,2$$

Rewriting Equations (23) and (28) by using Equation (30) as:

(31) $${\hat{P}}_{k|k}^{-1}=\sum_{i=1}^{2}{\left({\hat{P}}_{k|k}^{i}\right)}^{-1}-(l-1){\hat{P}}_{k|k-1}^{-1},l=2$$

(32) $${\hat{P}}_{k|k}^{-1}x_{v(k|k)}^{f}=\sum_{i=1}^{2}{\left({\hat{P}}_{k|k}^{i}\right)}^{-1}{\hat{x}}_{v(k|k)}^{i}-\left[\sum_{i=1}^{2}{\left({\hat{P}}_{k|k}^{i}\right)}^{-1}-{\hat{P}}_{k|k}^{-1}\right]{\hat{x}}_{v(k|k-1)}$$

Suppose that the initial values of state variable vector and covariance for fused and individual EKF SLAM are same, *i.e.*,

(33) $${\hat{x}}_{0|0}=x_{0|0}^{f}=x_{0|0}^{i}={\hat{x}}_{0|0}^{i},{\hat{P}}_{0|0}=P_{0|0}^{f}=P_{0|0}^{i}={\hat{P}}_{0|0}^{i}$$

and we also employ the assumptions listed in [30]:

(34) $$\begin{array}{l} {\hat{x}}_{k-1|k-1}=x_{k-1|k-1}^{f},{\hat{x}}_{k|k-1}=x_{k|k-1}^{f}\\ {\hat{P}}_{k-1|k-1}=P_{k-1|k-1}^{f},{\hat{P}}_{k|k-1}=P_{k|k-1}^{f} \end{array}$$

At step *k*, substituting Equation (34) into Equation (31), we have:

(35) $${\hat{P}}_{k|k}^{-1}={\left(P_{k|k-1}^{f}\right)}^{-1}+\sum_{i=1}^{2}{\left({\hat{P}}_{k|k}^{i}\right)}^{-1}-{\left(P_{k|k-1}^{f}\right)}^{-1}$$

Similar to Equation (19), we can get:

(36) $${\left({\hat{P}}_{k|k}^{i}\right)}^{-1}={\left({\hat{P}}_{k|k-1}^{i}\right)}^{-1}+{\left(H_{k}^{i}\right)}^{T}{\left(R_{k}^{i}\right)}^{-1}H_{k}^{i}\overset{\text{via}\;(31)}{=}{\left(P_{k|k-1}^{f}\right)}^{-1}+{\left(H_{k}^{i}\right)}^{T}{\left(R_{k}^{i}\right)}^{-1}H_{k}^{i}$$

Substituting Equation (36) into Equation (35), we obtain:

(37) $${\hat{P}}_{k|k}^{-1}={\left(P_{k|k-1}^{f}\right)}^{-1}+\sum_{i=1}^{2}{\left(H_{k}^{i}\right)}^{T}{\left(R_{k}^{i}\right)}^{-1}H_{k}^{i}$$

In comparing with Equation (23), we claim that :

(38) $${\hat{P}}_{k|k}^{-1}={\left(P_{k|k}^{f}\right)}^{-1}$$

On the other hand, with assumptions above we get the following equations by substituting Equation (18) into Equation (15):

(39) $${\left({\hat{P}}_{k|k}^{i}\right)}^{-1}{\hat{x}}_{v(k|k)}^{i}={\left({\hat{P}}_{k|k}^{i}\right)}^{-1}x_{v(k|k-1)}^{f}+{\left(H_{k}^{i}\right)}^{T}{\left(R_{k}^{i}\right)}^{-1}z_{k}-{\left(H_{k}^{i}\right)}^{T}{\left(R_{k}^{i}\right)}^{-1}h^{i}\left(x_{v(k|k-1)}^{i}\right)$$

and Equation (21) into Equation (20):

(40) $${\left(P_{k|k}^{f}\right)}^{-1}{\hat{x}}_{v(k|k)}={\left(P_{k|k}^{f}\right)}^{-1}x_{v(k|k-1)}^{f}+\sum_{i=1}^{2}\left[{\left(H_{k}^{i}\right)}^{T}{\left(R_{k}^{i}\right)}^{-1}z_{k}^{i}-{\left(H_{k}^{i}\right)}^{T}{\left(R_{k}^{i}\right)}^{-1}h^{i}\left(x_{v(k|k-1)}^{i}\right)\right]$$

With replacement of the related item in Equation (32) by Equation (39), and considering the conditions of Equation (34), the following derivation is obtained:

(41) $$\begin{array}{c} {\hat{P}}_{k|k}^{-1}x_{v(k|k)}^{f}={\left(P_{k|k}^{f}\right)}^{-1}x_{v(k|k-1)}^{f}+\sum_{i=1}^{2}\left[{\left(H_{k}^{i}\right)}^{T}{\left(R_{k}^{i}\right)}^{-1}z_{k}^{i}-{\left(H_{k}^{i}\right)}^{T}{\left(R_{k}^{i}\right)}^{-1}h^{i}\left(x_{v(k|k-1)}^{i}\right)\right]\\ \overset{\text{equation}\;(38)}{\to }{\left(P_{k|k}^{f}\right)}^{-1}x_{v(k|k)}^{f}={\left(P_{k|k}^{f}\right)}^{-1}x_{v(k|k-1)}^{f}+\sum_{i=1}^{2}\left[{\left(H_{k}^{i}\right)}^{T}{\left(R_{k}^{i}\right)}^{-1}z_{k}^{i}-{\left(H_{k}^{i}\right)}^{T}{\left(R_{k}^{i}\right)}^{-1}h^{i}\left(x_{v(k|k-1)}^{i}\right)\right] \end{array}$$

Comparing Equations (40) and (41), we assert that:

(42) $${\hat{x}}_{v(k|k)}=x_{v(k|k)}^{f}$$

It is obvious from Equations (38) and (42) that the performance of fused EKF SLAM does not change in the presence or absence of feedback. However, when the feedback is allowed into the individual EKF SLAM, the fused covariance of the state variable is decreased. This result is verified as follows: by Equations (19) and (37) we have the equation:

(43) $$\begin{array}{l} {\left(P_{k|k}^{i}\right)}^{-1}-{\left(P_{k|k-1}^{i}\right)}^{-1}={\left({\hat{P}}_{k|k}^{i}\right)}^{-1}-{\left(P_{k|k-1}^{f}\right)}^{-1}\Rightarrow \\ {\left({\hat{P}}_{k|k}^{i}\right)}^{-1}-{\left(P_{k|k}^{i}\right)}^{-1}={\left(P_{k|k-1}^{f}\right)}^{-1}-{\left(P_{k|k-1}^{i}\right)}^{-1}={\left(F_{k-1}P_{k|k-1}^{f}F_{k-1}^{T}+Q_{k}\right)}^{-1}-{\left(F_{k-1}P_{k|k-1}^{i}F_{k-1}^{T}+Q_{k}\right)}^{-1} \end{array}$$

It is easy to prove that Equation (43) is equal and larger than zero because $P_{k-1|k-1}^{f}\leq P_{k-1|k-1}^{i},k=2,3,...$ (cf. [30]). Therefore, we have ${\left({\hat{P}}_{k|k}^{i}\right)}^{-1}-{\left(P_{k|k}^{i}\right)}^{-1}={\left(P_{k|k-1}^{f}\right)}^{-1}-{\left(P_{k|k-1}^{i}\right)}^{-1}$, that is:

(44) $${\hat{P}}_{k|k}^{i}\leq P_{k|k}^{i},P_{k|k-1}^{f}\leq P_{k|k-1}^{i}$$

and also:

(45) $${\hat{P}}_{k|k}=P_{k|k}^{f}\leq P_{k|k}^{i}$$

which derives from Equations (19), (37) and (38) if and only if $\sum_{j\neq i}{\left(H_{r}^{j}\right)}^{T}{\left(R_{r}^{j}\right)}^{-1}H_{r}^{j}>0$, for some *r* ≤ *k* − 1. Please refer to [30] for this condition in detail.

It can be concluded that Equations (44) and (45) suggest that under a certain constraint the fused covariance of the state variable is reduced with the feedback. And when we use this fused state variables in SLAM, it will reduce the error of the localization and map features without changing the performance of individual EKF SLAM.

## References

[1] Davison A.J., Reid I.D., Molton N.D., Stasse O. (2007). MonoSLAM: Real-time single camera SLAM. Trans. Pat. Anal. Mach. Intell.

[2] Strasdat H., Montiel J.M.M., Davison A.J. Real-time monocular SLAM: Why filter?.

[3] Civera J., Davison A.J., Montiel J.M.M. Inverse depth to depth conversion for monocular SLAM.

[4] Eade E., Drummond T. Scalable monocular SLAM.

[5] Montiel J.M.M., Civera J., Davison A. Unified inverse depth parametrization for monocular SLAM.

[6] Eade E., Drummond T. Edge landmarks in monocular SLAM.

[7] Gee A.P., Mayol-Cuevas W. Real-time model-based SLAM using line segments.

[8] Smith P., Reid I., Davison A.J. Real-time monocular SLAM with straight lines.

[9] Lemaire T., Lacroix S. Monocular-vision based SLAM using line segments.

[10] Hartley R., Zisserman A. (2003). Multiple View Geometry in Computer Vision.

[11] Lowe D.G. (2004). Distinctive image features from scale-invariant keypoints. Int. J. Comput. Vis.

[12] Zhang X., Rad A., Wong Y.-K. (2008). A robust regression model for simultaneous localization and mapping in autonomous mobile robot. J. Intell. Robot. Syst.

[13] Sola J., Vidal-Calleja T., Devy M. Undelayed initialization of line segments in monocular SLAM.

[14] Folkesson J., Jensfelt P., Christensen H.I. Vision SLAM in the measurement subspace.

[15] Jeong W.Y., Lee K.M. Visual SLAM with line and corner features.

[16] Diosi A., Kleeman L. Advanced sonar and laser range finder fusion for simultaneous localization and mapping.

[17] Zhang G., Suh I.H. Building a partial 3D line-based map using a monocular SLAM.

[18] Kaess M., Dellaert F. (2009). Covariance recovery from a square root information matrix for data association. Robot. Auton. Syst.

[19] Margarita C., Andrew J.D. Active matching.

[20] Chli M., Davison A.J. (2009). Active matching for visual tracking. Robot. Auton. Syst.

[21] Kaess M., Dellaert F. (2010). Probabilistic structure matching for visual SLAM with a multi-camera rig. Comput. Vis. Image Underst.

[22] Booij O., Zivkovic Z., Kröse B. (2009). Efficient data association for view based SLAM using connected dominating sets. Robot. Auton. Syst.

[23] Kwon J., Lee K.M. Monocular SLAM with locally planar landmarks via geometric Rao-Blackwellized particle filtering on lie groups.

[24] Miro J.V., Dissanayake G., Weizhen Z. Vision-based SLAM using natural features in indoor environments.

[25] Sim R., Elinas P., Griffin M., Little J.J. Vision-based SLAM using the Rao-Blackwellised particle filter.

[26] Gil A., Reinoso O., Martinez Mozos O., Stachniss C., Burgard W. Improving data association in vision-based SLAM.

[27] Gil A., Reinoso O., Payá L., Ballesta M., Pedrero J.M. (2007). Managing data association in visual SLAM using SIFT features. Int. J. Fact. Autom. Robot. Soft Comput.

[28] Ahn S., Choi M., Choi J., Chung W.K. Data association using visual object recognition for EKF-SLAM in home environment.

[29] Chekhlov D., Mayol-Cuevas W., Calway A. Appearance based indexing for relocalisation in real-time visual SLAM.

[30] Zhu Y. (2003). Multisensor Decision and Estimation Fusion.

[31] Wijesoma W.S., Perera L.D.L., Adams M.D. (2006). Toward multidimensional assignment data association in robot localization and mapping. IEEE Trans. Robot.

